# Directionality eclipses agency: How both directional and social cues improve spatial perspective taking

**DOI:** 10.3758/s13423-021-01896-y

**Published:** 2021-03-25

**Authors:** Peri Gunalp, Elizabeth R. Chrastil, Mary Hegarty

**Affiliations:** 1 Department of Psychological and Brain Sciences, University of California Santa Barbara, Santa Barbara, CA 93106 USA; 2 Department of Neurobiology & Behavior; Center for the Neurobiology of Learning and Memory, University of California Irvine, Irvine, CA USA

**Keywords:** Spatial perspective taking, Social cues, Directional cues, Perspective shift, Mental transformation, Spatial cognition, Embodied cognition

## Abstract

Research on spatial perspective taking has suggested that including an agent in the display benefits performance. However, little research has examined the mechanisms underlying this benefit. Here, we examine how an agent benefits performance by examining its effects on three mental steps in a perspective-taking task: (1) imagining oneself at a location (station point) within in the array, (2) adopting a different perspective (heading), and (3) pointing to an object from that perspective. We also examine whether a non-agentive directional cue (an arrow) is sufficient to improve performance in an abstract map-like display. We compared a non-directional cue to two cues for position and orientation: a human figure (agentive, directional) and an arrow (non-agentive, directional). To examine the effects of cues on steps 2 and 3 of the perspective-taking process, magnitude of the initial perspective shift and pointing direction were varied across trials. Response time and error increased with the magnitude of the imagined perspective shift and pointing to the front was more accurate than pointing to the side, or back, but these effects were independent of directional cue. A directional cue alone was sufficient to improve performance relative to control, and agency did not provide additional benefit. The results overall indicate that most people adopt an embodied cognition strategy to perform this task and directional cues facilitate the first step of the perspective-taking process, imagining oneself at a location within in the array.

## Introduction

Spatial perspective taking is the process of imagining how an object or scene would appear from a viewpoint other than one’s current physical perspective. It is important for numerous cognitive processes, including understanding the layout of an environment (Fields & Shelton, 2006), navigation (e.g., Holmes et al., 2017), and giving directions (Hegarty & Waller, 2004). Extant research has illustrated the developmental trajectory of this skill (Epley et al., 2004; Newcombe & Frick, 2010), sex differences (Lawton, 1994; Linn & Petersen, 1985; Tarampi et al., 2016) and individual differences in performance (Hegarty & Waller, 2004; Kozhevnikov & Hegarty, 2001), and has connected perspective taking to other skills like empathy (e.g., Ruby & Decety, 2004), mental simulation, and embodied cognition (e.g., Kessler & Wang, 2012).

**Fig. 1:**
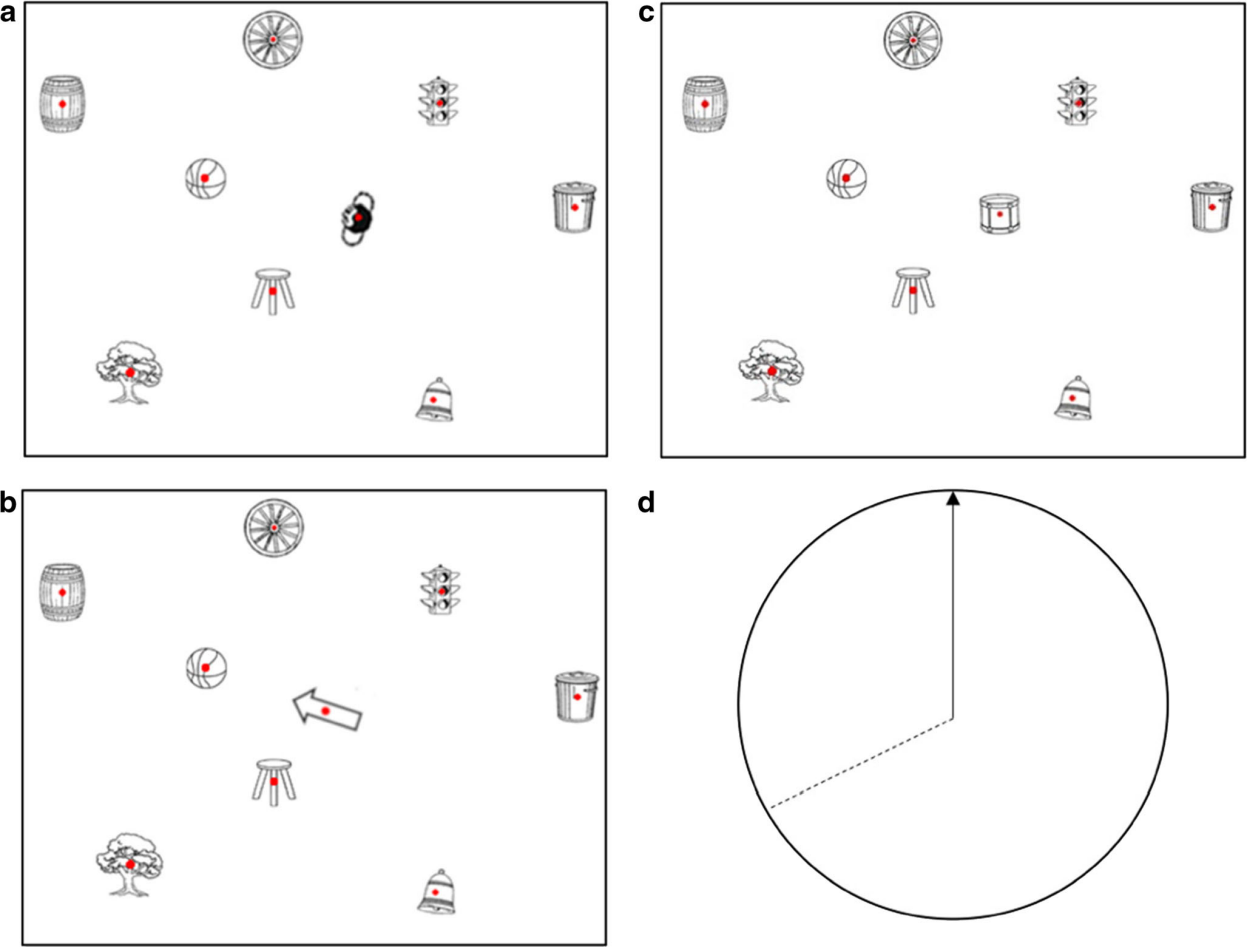
Three arrays used in the current research: (**A**) human figure, (**B**) arrow, and (**C**) control. Also pictured is the arrow circle on which participants input their pointing judgments (**D**)

The Spatial Orientation Test (SOT; Hegarty & Waller, 2004; Kozhevnikov & Hegarty, 2001) is a common measure of spatial perspective-taking ability. On each trial of the SOT, participants are asked to imagine standing at one object (station point) in a map-like array, facing a second object, and then to point to a third (target) object (see Fig. 1).

Tarampi et al. (2016) found that the inclusion of a human figure in the task array of the SOT improved performance relative to a control array. This supports earlier research on the influence of agency on perspective-taking performance (Clements-Stephens et al., 2013; Shelton et al., 2012). In a follow-up study, Gunalp et al. (2019) compared the effects of both an arrow and a human figure in an immersive desktop Virtual Reality (VR) version of the SOT. In contrast with a human figure, an arrow provides a directional cue by facing the correct direction on each trial, and provides a consistent station point, but is inanimate (or non-agentive). In this study, a human figure improved performance compared to an arrow, which did not differ from control. This result was interpreted as indicating an embodied cognition process (e.g., Kessler & Rutherford, 2010). Participants reported using mental simulation strategies that entailed imagining of themselves in the array to make pointing estimates and Gunalp et al. concluded that including a human figure in the display facilitated this process.

The major aim of the present study is to examine how different directional cues affect perspective-taking performance. Previous research has identified three steps in the SOT perspective-taking task: (1) initial identification of the station point (location in the array to be assumed), (2) imagining a new facing direction, and (3) making pointing judgments (Hegarty, & Kozhevnikov, 1999). This study addresses whether and how social and directional cues affect steps 1, 2, and 3 of the perspective-taking process. While it is likely that directional and agentive cues affect initial identification of the station point (step 1), they might also affect steps 2 and 3. This could not be determined from previous studies (Gunalp et al., 2019; Tarampi et al., 2016) as those studies only examined performance collapsed over the 12 items in the SOT, which do not systematically vary initial perspective shift (step 2) and pointing direction (step 3). Here we systematically varied these trial features to examine the effects of cues on steps 2 and 3.

A second aim of the present study was to examine whether the effects of social versus directional cues found by Gunalp et al. generalize to the abstract map-type display used in the SOT psychometric test. Gunalp et al. used a more naturalistic environment than the original SOT (and Tarampi et al., 2016), namely a three-dimensional immersive virtual environment, viewed through a head-mounted display, showing a park. It is possible that the human figure improved performance relative to an arrow because a three-dimensional human figure is a more natural cue in this environment than an arrow, and is easier to embody than an arrow or the other inanimate objects in the task array. Further, the viewing angle on the array of objects was oblique (130°), which contrasts the over-head view (180°) of the map-like array in the SOT in the present experiments, and it may be more difficult to perceive the direction of an arrow in an oblique view. Therefore we cannot assume that the findings of Gunalp et al. regarding social versus directional cues will generalize to the map-like SOT display.

**Fig. 2:**
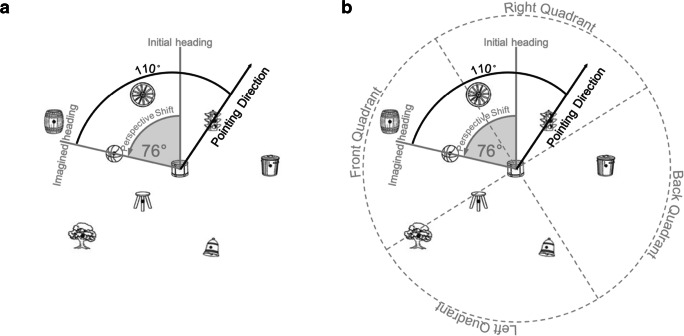
Graphic depiction of both imagined perspective shift and pointing quadrant for special orientation test (SOT) trials. In this example, the trial asked participants to imagine standing at the drum facing the basketball, then to point to the traffic light. If the initial heading is straight up, reflecting the participant seated at the computer looking at the array, it takes a 76° shift in perspective to face the basketball. From that new perspective, the traffic light is 110° behind and to the right. Panel A represents step 2 of the perspective-taking process, and panel B represents step 3

Trials in the SOT vary both in the magnitude of the imagined shift in perspective (the difference between one’s actual heading and the heading to be imagined) and in the direction of pointing to the target (in front, to the right or left, or behind) (Fig. 2). Previous research shows that pointing error on the SOT is greater after a larger imagined perspective shift (Kozhevnikov & Hegarty, 2001). These findings, which reflect step 2 of perspective taking, suggest that perspective taking is an analog transformation (Rieser, 1989) like mental rotation (Shepard & Metzler, 1971), with the added difficulty of inhibiting one’s current perspective (May, 2004), especially when making a pointing response that involves a conflict between one’s physical and imagined perspectives (de Vega & Rodrigo, 2001; Wraga, 2003). In perspective-taking tasks, individuals report mentally simulating being at a location and facing a direction in the array, and performing transformations relative to their new perspective (Kozhevnikov & Hegarty, 2001; see also Gunalp et al., 2019; Zacks & Michelon, 2005; Zacks, Mires, Tversky, & Hazeltine, 2000). It is plausible that a social or directional cue might facilitate the process of assuming a different perspective or inhibiting one’s current perspective, in which case we would expect a reduced effect of perspective shift on pointing error in these cue conditions.

Step 3 of the perspective-taking process – making pointing judgments – may also be an analog process, with increases in time or error as the direction of the target deviates more from the imagined perspective. However, if perspective taking is accomplished by an embodied cognition process, an alternative is that this step of the process is influenced by the constraints of the human body. In this respect, some research indicates that pointing to a target in front of or behind the imagined heading is easier than pointing to an object to the right or left (e.g., Franklin et al., 1995; Franklin & Tversky, 1990; Franklin et al., 1992; Hintzman, O’Dell, & Arndt, 1981; Sholl, 1987; Werner & Schmidt, 2000), and other research suggests that pointing in front is easier than pointing behind (Horn & Loomis, 2004; Shelton & McNamara, 2001). Both of these findings were evident in preliminary studies of the SOT (Kozhevnikov & Hegarty, 2001). We might expect that pointing would be more influenced by the constraints of the human body when the cue is a human figure, rather than an arrow or other cue.

### Hypotheses

First, we investigated alternative hypotheses about which steps of the perspective-taking process are facilitated by a human figure or an arrow. If these cues affect step 1 alone, this should be evident in a main effect of cue. If they affect steps 2 and 3, this should be evident in interactions between cue type and perspective shift (for step 2) and cue type and pointing direction (for step 3).

Second, we contrasted two alternative hypotheses regarding the effects of a cue on the map-like display used in the SOT: the agency hypothesis and the directionality hypothesis. According to the first hypothesis, agency of the cue is necessary to facilitate performance, so a human figure should be associated with faster and more accurate performance relative to both an arrow and a control array. An interaction between cue presence and cue type would indicate a difference between the human figure and arrow. Alternatively, if a directional cue is sufficient to improve performance in a map-like display (directionality hypothesis), then performance should be improved in both the human figure and arrow conditions relative to control.

As in previous research (Hegarty & Kozhevnikov, 1999), we expected greater angular error and longer reaction times with larger perspective shifts, because they present more conflict between physical and imagined frames of reference and because the response was a pointing response (cf. de Vega & Rodrigo, 2001; Wraga, 2003). We also predicted that pointing to the front of the imagined position would be faster or more accurate, either because pointing is an analog process or because imagined pointing is affected by constraints of the human body; alternatively pointing to both the front and back might be facilitated because of relation to the axes of the human body.

## Experiment 1

### Method

#### Participants

Eighty participants from the University of California, Santa Barbara participated in this study for course credit. Five (three women, two men) participants were excluded due to error above chance levels, suggesting that they did not understand the task. Of the remaining participants 37 (21 women, 16 men) were assigned to the arrow condition and 38 participants (18 women, 20 men) were assigned to the human figure condition. Participants were aged 17–22 years (*M* = 18.47, *SD* = .88). A power analysis for ANOVA was conducted using G*Power with an alpha level of .05 and power of .80, indicating that a minimum sample size of 72 would be needed. The present sample size exceeds this minimum.

#### Design

This experiment employed a mixed factor design with cue presence (two levels: control [no cue] vs. directional cue), absolute value of initial perspective shift (four levels: 0–45°, 45–90°, 90–135°, 135–180°) and pointing quadrant (four levels: left, right, back, front) manipulated within subjects. Type of directional cue (arrow vs. human figure) in the directional cue condition was manipulated between subjects, while all participants performed the control task (thus controlling for any sampling error between the groups). Task order was counterbalanced between subjects such that there were four groups who completed the task as follows: control-human figure, human figure-control, control-arrow, arrow-control. Absolute angular error and response time were measured as dependent variables.

#### Materials and apparatus

This study employed a computerized perspective-taking task similar to the computerized Spatial Orientation Test (SOT) recently developed by Friedman, Kohler, Gunalp, Boone, and Hegarty (2019). This task was displayed on Dell 24-in. P24124 (60-Hz refresh rate) monitors with Nvidia GeForce GTX (660) graphics cards. The computerized task was displayed through E Prime (2.0, Schneider, Eschman, & Zuccolotto, 2012). As in earlier versions of this task, the display included an array of objects and an arrow circle in which participants reported their direction estimates. The array contained nine non-directional objects that do not have a clear front or back or facing direction (Fig. 1). The arrow circle contained a vertical arrow indicating the standing position (station point) and facing direction/imagined heading with written object labels for each trial. On each trial, participants were asked to imagine standing at one object in the array, facing a second, and then to point to a third. In the control array condition, a trial might read: “Imagine you are standing at the bell facing the tree. Point to the drum.” In the human figure array condition, a trial might read: “Take the perspective of the person facing the tree. Point to the drum” (see Fig. 1A). Trials for the arrow array read: “Imagine you are standing at the arrow, facing the tree, point to the drum” (see Fig. 1B). The instructions differed between display types for two reasons: to maximize the potential effect of the human figure in the display, and to be as intuitive as possible for participants (being told to take the perspective of an arrow is unusual).

Participants completed 32 test trials in each condition that varied the magnitude of the initial perspective shift and the pointing quadrant. Initial perspective shift was categorized into four distinct bins with 45° increments (0–45°, 45–90°, 90–135°, etc.), collapsed over clockwise versus counterclockwise perspective shifts for analyses and graphing such that 0–45° and 315–360° were grouped, etc. Pointing quadrants were categorized as front, back, right, or left. The front quadrant encompassed 45° clockwise and counterclockwise, and the right quadrant encompassed 45–135°, etc. For each pointing quadrant there were eight trials. Right and left pointing directions were collapsed in the analysis. Thus there were four levels of perspective shift by three levels of pointing direction.

An online questionnaire was used to collect demographic information and self-reports of participants’ strategies for solving the perspective-taking tasks. Participants were asked to choose between four strategies identified in previous research (Kozhevnikov & Hegarty, 2001): (1) imagining being in the array and rotating to the indicated heading, (2) imagining the angle created by the objects from the indicated viewpoint within the array, (3) superimposing the array on the arrow circle, and (4) superimposing the arrow circle on the array. The first two strategies were categorized as involving mental simulation, and the third and fourth strategies were categorized as abstract. Participants were given an opportunity to describe their own strategy if it was different from one of the given strategies.

#### Procedure

Participants were run in groups of one to three, and after giving informed consent began the first perspective-taking task (SOT) with one of the arrays (control, arrow, or human figure). Half of the participants completed the control SOT first, and half completed a directional SOT first. If participants completed the tasks in a group, all completed the same order of tasks. The experimenter read the instructions displayed on the computer aloud while participants followed along. After the instructions, participants practiced how to respond, and then completed three practice trials with feedback before proceeding to the 32 test trials. After completing one version of the task, participants were given the instructions for the second version and completed that task. Finally they completed the online questionnaire.

## Results

Angular error data for this experiment were positively skewed, and were log-transformed for subsequent analyses. Response-time data were normally distributed.^1^ An alpha level of .025 was adopted for all analyses, as there were two dependent measures.

### Perspective shift

We first analyzed the data according to size of perspective shift required (step 2 of perspective taking), collapsing over pointing direction.

#### Angular error

**Fig. 3:**
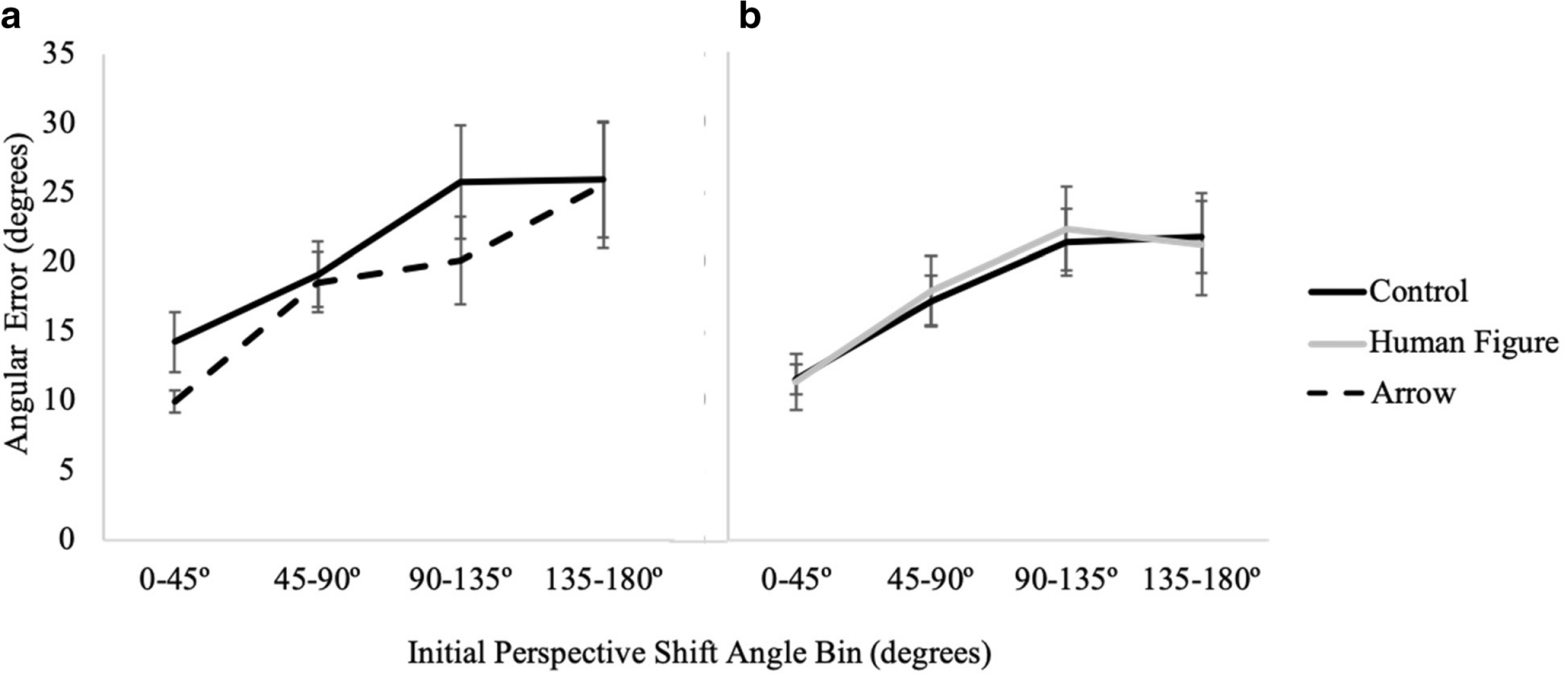
Results from Experiment 1 showing absolute angular error as a function of cue type and initial perspective shift angle bin for (**A**) control vs. arrow and (**B**) control vs. human figure conditions

A 2 (cue presence: control, directional) × 4 (perspective shift absolute value: 0–45°, 45–90°, 90–135°, 135–180°) × 2 (cue type: arrow, human figure) mixed factors repeated-measures ANOVA with post hoc (Bonferroni) pair-wise comparisons, corrected for multiple comparisons, revealed a significant main effect of perspective shift, *F*(3, 219) = 46.30, *p <* .001, *η*_*p*_^*2*^ = .39 (see Fig. 3). No other effects or interactions were significant, *p*s > .16 (see Table 1). Notably there were no significant effects of cue presence, cue type, or their interaction. Mean angular error (log transformed) was 2.26 (*SE* = .06) in the combined control conditions, 2.22 (*SE* = .09) in the arrow condition and 2.20 (*SE* = .09) in the human figure conditions.

**Table 1:** Significance level (p-values) and effect sizes (partial eta-squared) for each effect in the analyses of variance (significant effect indicted in bold type) for Experiments 1 and 2

	Experiment 1		Experiment 2	
	Angular error p (*η*_*p*_^*2*^)	Response time p (*η*_*p*_^*2*^)	Angular error p (*η*_*p*_^*2*^)	Response time p (*η*_*p*_^*2*^)
Perspective shift (0–45°, 45–90°, 90–135°,135–180°) (data collapsed over pointing quadrant)				
Main effects:				
Perspective shift	**.001 (.39)**	**.001 (.42)**	**.001 (.40)**	**.001 (.38)**
Presence of directional cue (directional vs. control)	.23 (.02)	**.001 (.16)**	.001 (.31)*	**.001 (.30)**
Type of directional cue (human vs. arrow)	.68 (.00)	.38 (.01)	.54 (.01)	.89 (.00)
Interactions:				
Type of directional cue*presence of directional cue	.58 (.00)	.95 (.00)	.03 (.06)	.37 (.01)
Perspective shift*type of directional cue	.16 (.02)	.23 (.02)	1.0 (.00)	.15 (.02)
Perspective shift * presence of directional cue	.47 (.01)	.62 (.01)	.16 (.02)	.52 (.01)
Perspective shift*directional cue * type of directional cue	.72 (.01)	.61 (.01)	.24 (.02)	.98 (.00)
Pointing quadrant (front, back, right, left) (data collapsed over perspective shift)				
Main effects:				
Pointing quadrant	**.001 (.60)**	**.001 (.45)**	**.001 (.55)**	**.001 (.47)**
Presence of directional cue (directional vs. control)	.48 (.01)	**.001 (.15)**	.001 (.23)*	**.001 (.27)**
Type of directional cue (human vs. arrow)	.25 (.02)	.35 (.01)	.73 (.001)	.98 (.00)
Interactions:				
Type of directional cue*presence of directional cue	.33 (.01)	.86 (.00)	.06 (.05)	.40 (.01)
Pointing quadrant *type of directional cue	.31 (.02)	.12 (.03)	.41 (.01)	.13 (.03)
Pointing quadrant*presence of directional cue	.74 (.00)	.70 (.05)	.29 (.02)	.04 (.04)
Pointing quadrant*directional cue * type of directional cue	.36 (.70)	.36 (.01)	.51 (.01)	.96 (.00)

An asterisk indicates that results differ between Experiments 1 and 2. An alpha level of .025 was adopted as there were two dependent variables (angular error and response time)

#### Response time

**Fig. 4:**
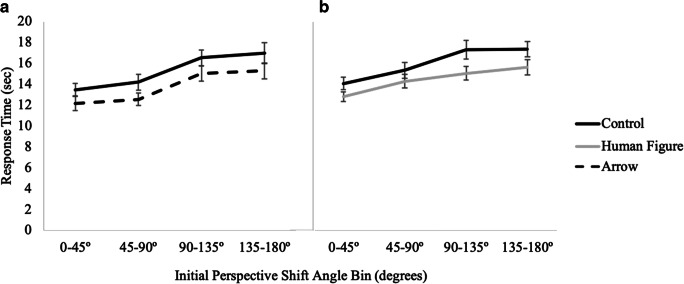
Results from Experiment 1 showing response time as a function of cue type and magnitude of initial perspective shift for (**A**) control vs. arrow and (**B**) control vs. human figure

A 2 (cue presence) × 4 (perspective shift) × 2 (cue type: arrow, human figure) mixed factors repeated-measures ANOVA indicated a main effect of cue presence, *F*(1, 73) = 14.05, *p <* .001, *η*_*p*_^*2*^ = .16, with post hoc pairwise comparisons indicating that participants were significantly faster with the directional arrays (*M* = 14.10, *SE* = .40) than with the control array (*M* = 15.65, *SE* = .49). There was also a significant main effect of perspective shift, *F*(3, 219) = 52.96, *p <* .001, *η*_*p*_^*2*^ = .42 (see Fig. 4). Notably, there was no interaction of cue presence with cue type, and a planned comparison indicated that response times did not significantly differ for the arrow (*M* = 13.76, *SE* = .61) and human figure (*M* = 14.44, *SE* = .52) conditions, *t*(73) = .84, *p* = .41. No other effects or interactions were significant, *p*s > .23 (see Table 1).

### Pointing quadrant

We also analyzed the data based on the direction of pointing (pointing quadrant) for each trial (step 3 of perspective taking) collapsing over perspective shift. Pointing quadrants left and right were collapsed for these analyses because the current work does not make any predictions about differences between pointing to the left or right.

#### Angular error

**Fig. 5:**
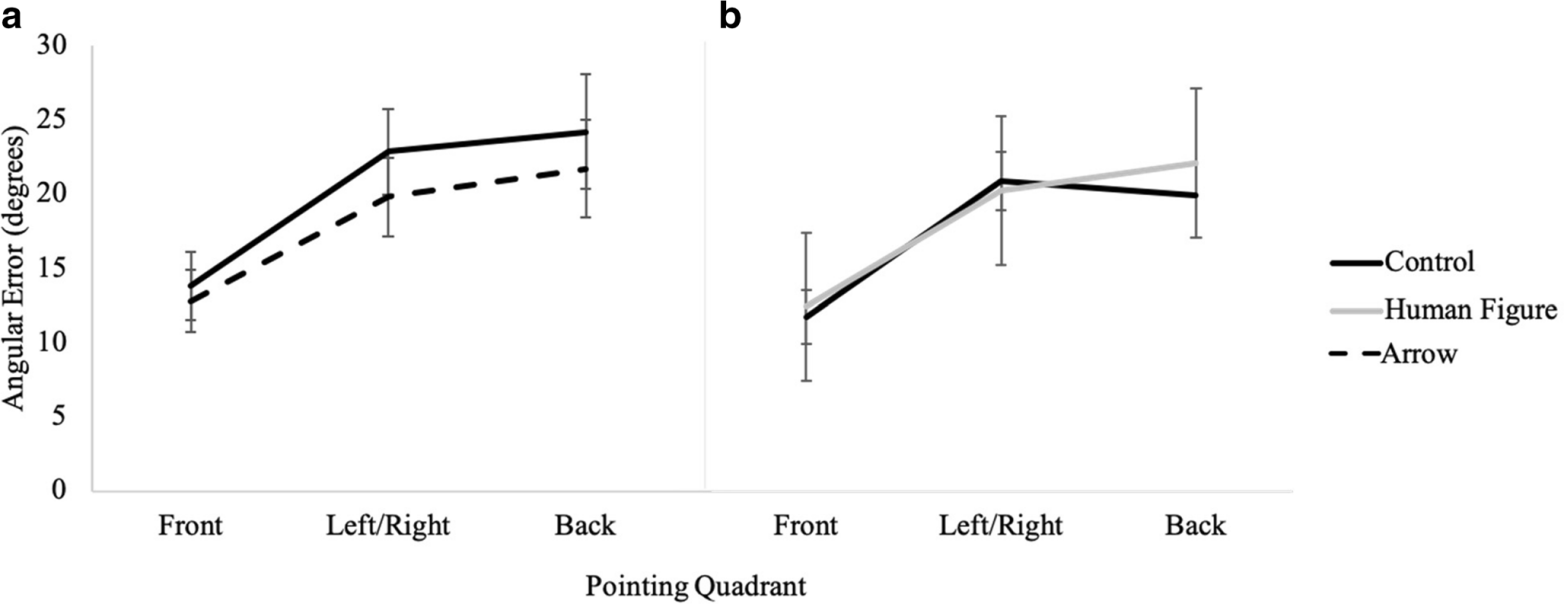
Results from Experiment 1 showing absolute angular error as a function of cue type and pointing quadrant for (**A**) control vs. arrow and (**B**) control vs. human figure

A 2 (cue presence) × 3 (pointing quadrant: front, left/right, back) × 2 (cue type: arrow, human figure) mixed factors repeated-measures ANOVA revealed a significant main effect of pointing quadrant, *F*(2, 73) = 109.12, *p <* .001, *η*_*p*_^*2*^ = .60. Post hoc pairwise comparisons indicated that participants had significantly less error in pointing to the front quadrant (*M* = 1.86, *SE* = .05) than to the left or right (*M* = 2.40, *SE* = .05), and to the back (*M* = 2.32, *SE* = .06), but left/right was not significantly different from back (see Fig. 9). No other effects or interactions were significant, *p*s > .31 (see Table 1) Fig. 5.

#### Response time

**Fig. 6:**
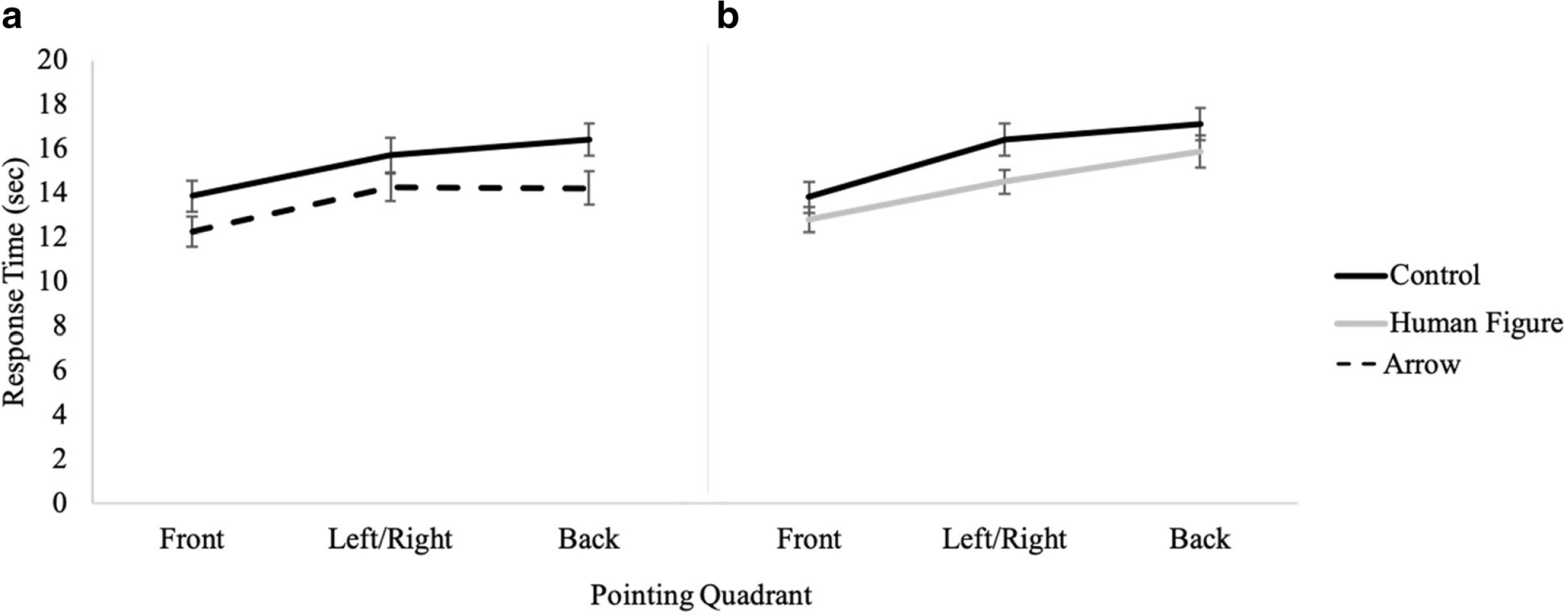
Results from Experiment 1 showing response time as a function of cue type and pointing quadrant for (**A**) control vs. arrow and (**B**) control vs. human figure

A 2 (cue presence) × 3 (pointing quadrant) × 2 (cue type) mixed factors repeated-measures ANOVA revealed a significant main effect of cue presence, *F*(1, 73) =12.80, *p <* .001, *η*_*p*_^*2*^ = .15, such that participants were significantly faster with the directional array (*M* = 14.00, *SE* = .41) than with the control array (*M* = 15.52, *SE* = .47). There was also a significant main effect of pointing quadrant, *F*(2, 73) = 59.25, *p <* .001, *η*_*p*_^*2*^ = .45, with post hoc pairwise comparisons indicating that participants were significantly faster for pointing to the front quadrant (*M* = 13.17, *SE* = .39) than to the left and right (*M* = 15.21, *SE* = .43), and pointing to the left or right was significantly faster than pointing to the back (*M* = 15.90, *SE* = .43) quadrants (see Fig. 6). No other effects or interactions were significant (see Table 1).

### Discussion

Experiment 1 indicated that a directional cue increases speed of response on the SOT perspective-taking task and does not differ from a social cue. However, the instructions differed between the human figure and arrow cues. The human figure instructions told participants to “take the perspective of the person facing the tree,” whereas the arrow instructions told participants to “imagine you are standing at the arrow facing the tree.” A second study was conducted using the same instructions with all cues to address this confound.

## Experiment 2

### Participants

Eighty-four participants from the University of California, Santa Barbara participated in this study for course credit. One participant (a woman) was excluded for missing data. Of remaining participants (mean age 19.25 years, *SD* = 1.28 ), 38 (23 women, 15 men) were assigned to the arrow condition and 45 (28 women, 17 men) were assigned to the human figure condition.

### Design, materials, and apparatus

The present experiment followed the same design as Experiment 1. The materials were identical to those of Experiment 1, apart from the instructions for the human figure array. In this experiment, the instructions for the human figure cue type read: “Imagine you are standing at the person, facing the tree, point to the ball,” rather than: “Take the perspective of the person, facing the tree, point to the ball” (which was used in Experiment 1).

### Procedure

The procedure for this experiment was identical to that of Experiment 1.

## Results

As in Experiment 1, angular error data were non-normally distributed, so subsequent analyses were conducted on log-transformed data. Response-time data were normally distributed, and raw data were analyzed.^2^

### Perspective shift

#### Angular error

**Fig. 7:**
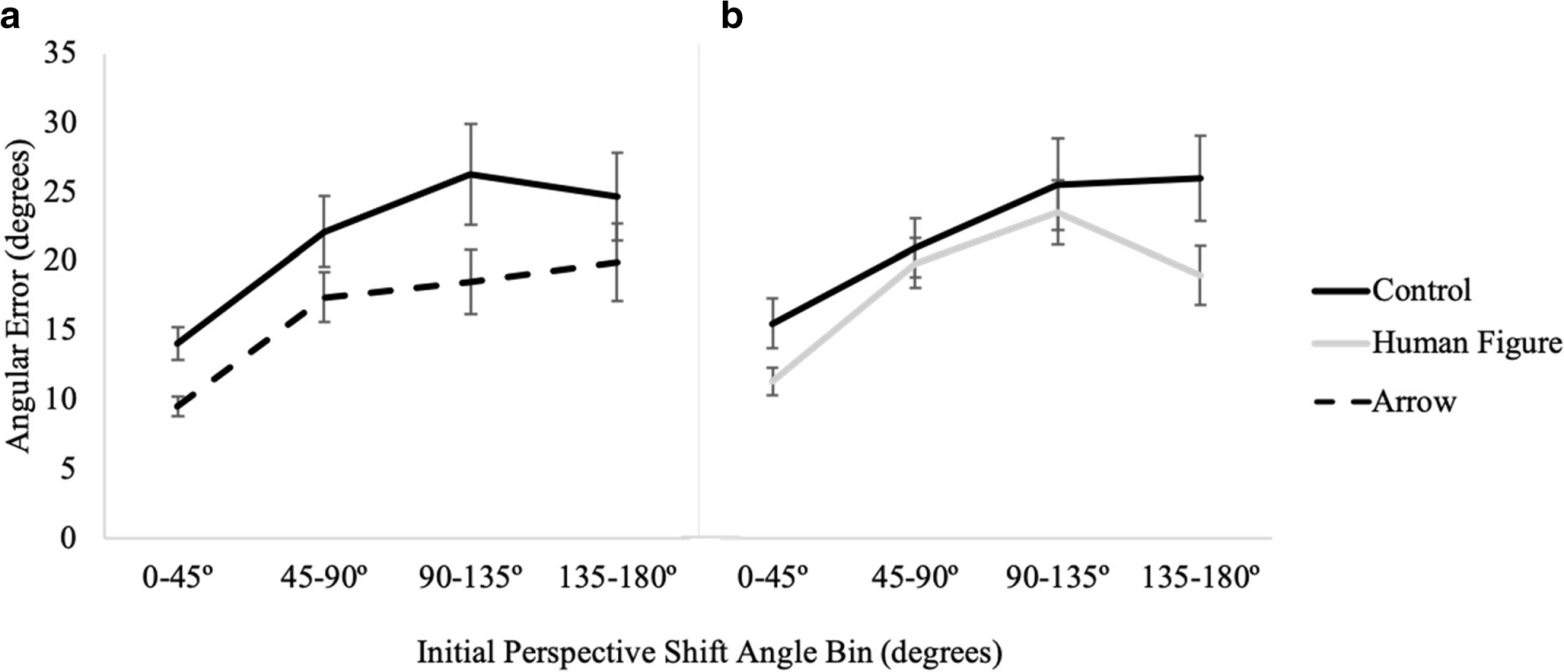
Results from Experiment 2 showing accuracy as a function of cue type and perspective shift for (**A**) control vs. arrow and for (**B**) control vs. human figure

A 2 (cue presence: control, directional) × 4 (perspective shift absolute value: 0–45°, 45–90°, 90–135°, 135–180°) × 2 (cue type: arrow, human figure) mixed model ANOVA revealed that participants were significantly more accurate in the directional conditions (*M* = 2.1, *SE* = .04) than in the control condition (*M* = 2.3, *SE* = .05, *F* (1,81) = 36.22, *p* < .001, *η*_*p*_^*2*^ = .31. As in Experiment 1, there was also a significant main effect of perspective shift, *F* (3,243) = 53.22, *p* < .001, *η*_*p*_^*2*^ = .40 (see Fig. 7). There was a marginally significant interaction of cue presence and directional cue type, *F* (1,81) = 4.67, *p* = .03, *η*_*p*_^*2*^ = .06, indicating, if anything, that there was a larger difference in performance between the control and arrow conditions than the control and human figure conditions. While there was no difference between the human figure (*M* = 2.15, *SE* = .06) and the arrow (*M* = 2.14, *SE* = .07) conditions, *p* = .90, these groups differed somewhat on the control task, with higher angular errors in the control for those assigned to the arrow condition (*M* = 2.39, *SE* = .08) than to the human figure condition (*M* = 2.27, *SE* = .06). No other effects or interactions were significant, *p*s > .16 (see Table 1).

#### Response time

**Fig. 8:**
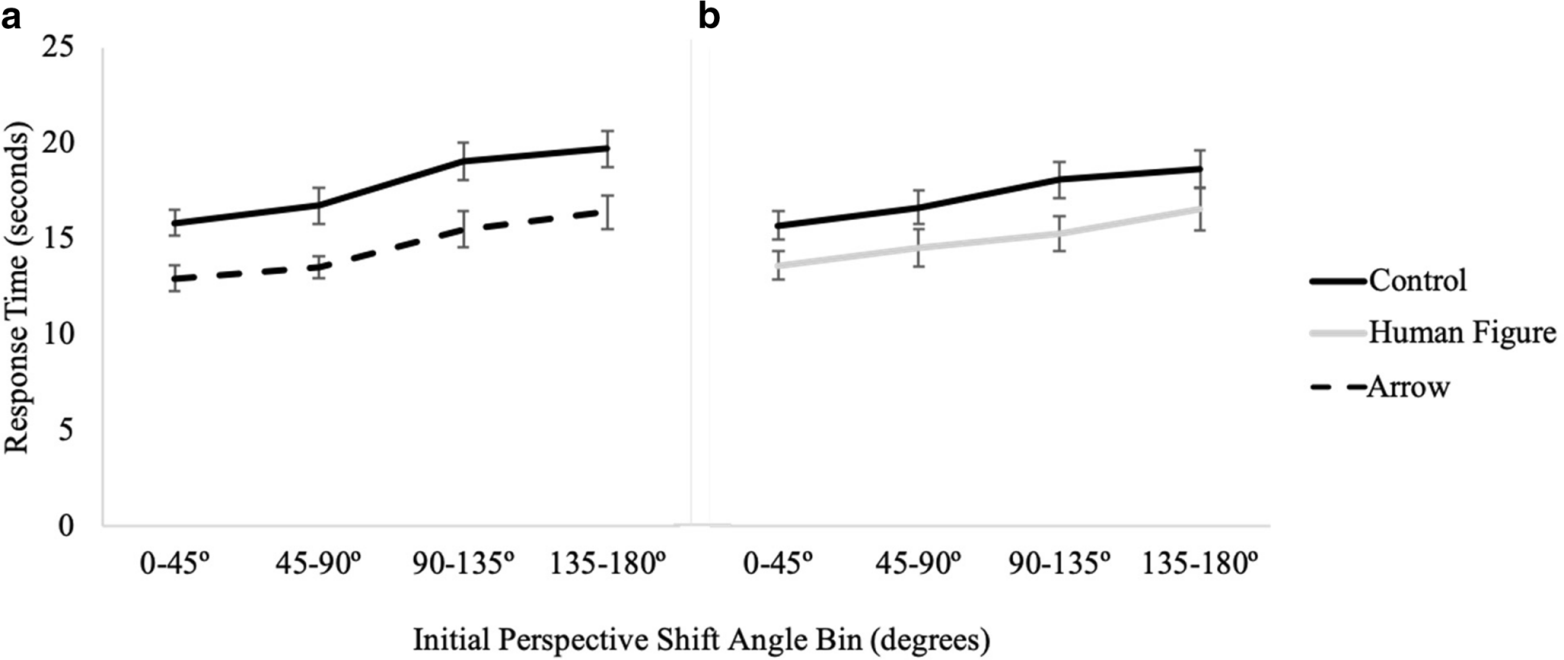
Results from Experiment 2 showing response time as a function of cue type and perspective shift for (**A**) control vs. arrow and for (**B**) control vs. human figure

A 2 (cue presence) × 4 (perspective) × 2 (cue type: arrow, human figure) mixed factors repeated-measures ANOVA revealed a main effect of cue presence, *F*(1, 81) = 34.19, *p <* .001, *η*_*p*_^*2*^ = .30, such that participants were significantly faster in the directional conditions (*M* = 14.9, *SE* = .6) than in the control condition (*M* = 17.5, *SE* = .6). A planned comparison (independent-samples t-test) indicated that there was no difference between the human figure (*M* = 14.98, *SE* = .88) and the arrow (*M* = 14.72, *SE* = .71) conditions, *t*(81) = .23, *p* = .82. Again, there was also a significant main effect of perspective shift, *F*(3, 243) = 49.63, *p <* .001, *η*_*p*_^*2*^ = .38 (see Fig. 8). No other effects or interactions were significant, *p*s > .15 (see Table 1).

### Pointing quadrant

#### Angular error

**Fig. 9:**
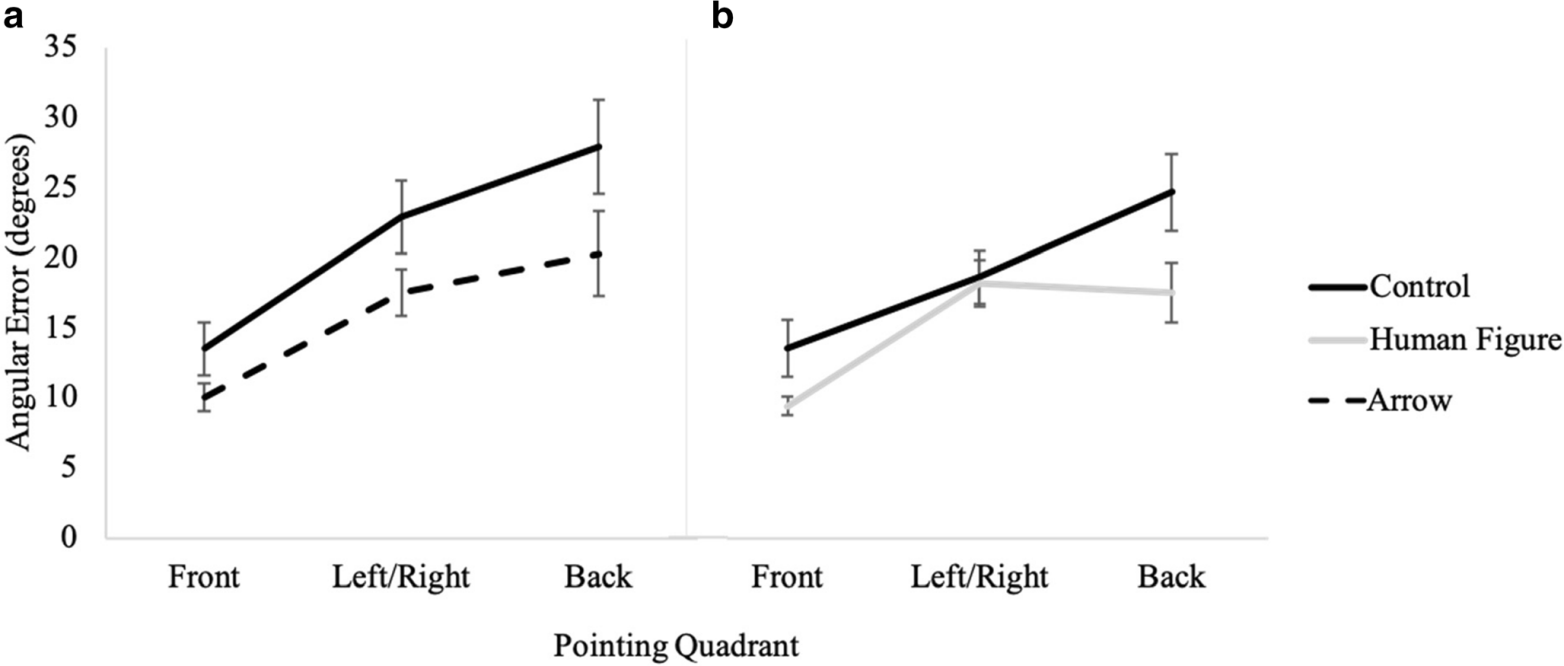
Results from Experiment 2 showing accuracy as a function of cue type and pointing quadrant for (**A**) control vs. arrow and for (**B**) control vs. human figure

A 2 (cue presence: control, directional) × 3 (pointing quadrant: front, left/right, back) × 2 (cue type: arrow, human figure) mixed factors repeated-measures ANOVA revealed that participants had significantly less error in the directional conditions (*M* = 2.13, *SE* = .05) than in the control condition (*M* = 2.30, *SE* = .05; *F*(1, 82) = 23.76, *p <* .001, *η*_*p*_^*2*^ = .23). This ANOVA also revealed a significant main effect of pointing quadrant, *F*(2, 81) = 96.83, *p <* .001, *η*_*p*_^*2*^ = .55, such that participants had significantly less error when pointing to the front quadrant (*M* = 1.87, *SE* = .05) than to the left/right (*M* = 2.36, *SE* = .05), and to the back (*M* = 2.41, *SE* = .06), but left/right was not significantly different from back (see Fig. 9). No other effects or interactions were significant, *p*s > .06 (see Table 1).

#### Response time

**Fig. 10:**
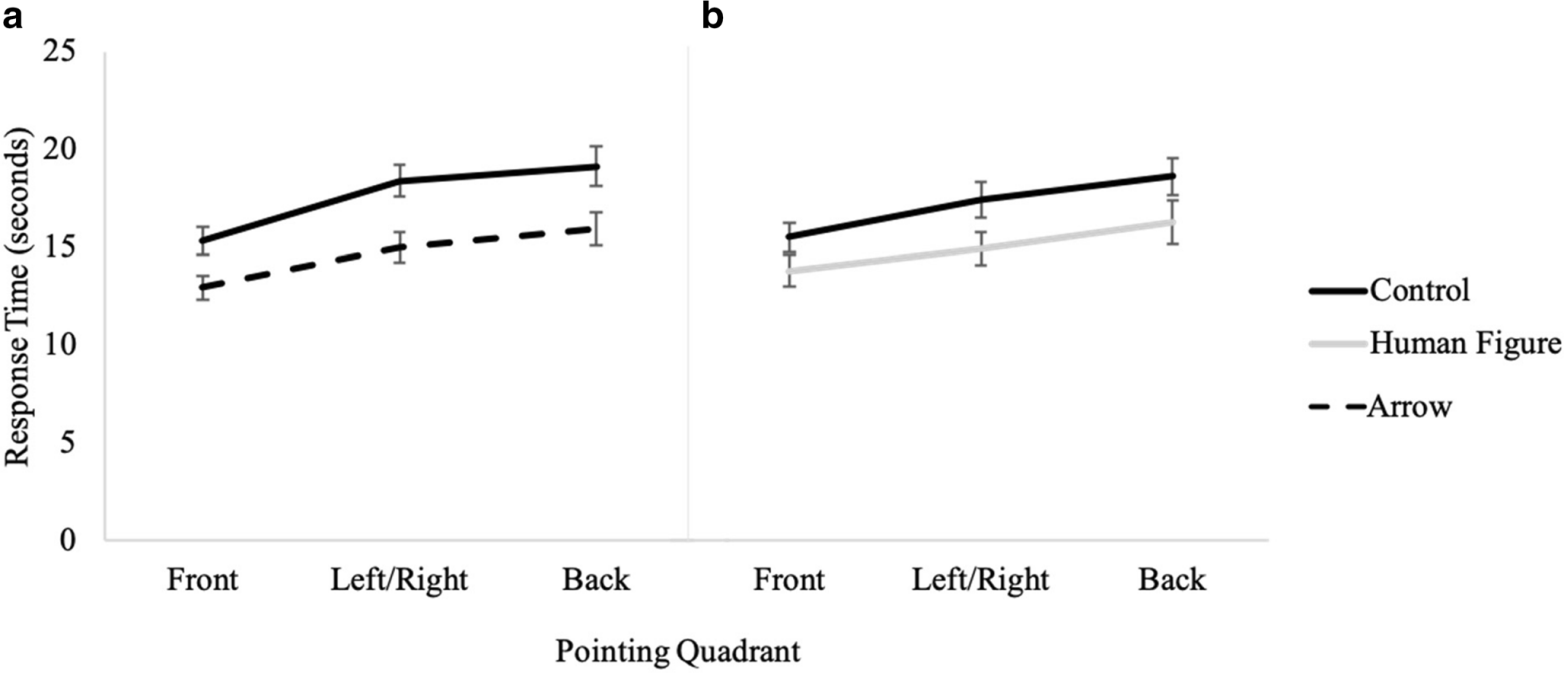
Results from Experiment 2 showing response time as a function of cue type and pointing quadrant for (**A**) control vs. arrow and for (**B**) control vs. human figure

A 2 (cue presence) × 3 (pointing quadrant) × 2 (cue type: arrow, human figure) mixed model ANOVA revealed that participants were significantly faster in the directional conditions (*M* = 14.81, *SE* = .59) than in the control condition (*M* = 17.42, *SE* = .58; *F*(1, 81) =30.32, *p <* .001, *η*_*p*_^*2*^ = .27). There was also a significant main effect of pointing quadrant, *F*(2, 81) = 72.70, *p <* .001, *η*_*p*_^*2*^ = .47, such that participants were significantly faster for pointing to the front quadrant (*M* = 14.40, *SE* = .46) than to the left/right (*M* = 16.44, *SE* = .55), both of which were significantly faster than back (*M* = 17.51, *SE* = .63) (see Fig. 10). The interaction of presence of directional cue and pointing quadrant was marginally significant, *F*(2, 81) = 3.18, *p =* .04, *η*_*p*_^*2*^ = .04, such that the difference between pointing to the front and the side was greater for the control condition than for the directional conditions. No other effects or interactions were significant, *p*s > .13 (see Table 1).

#### Self-reported strategies

Survey data from Experiments 1 and 2 indicated that most participants in all conditions (control, arrow, and human figure) reported using a mental simulation strategy rather than an abstract strategy (see Table 2). There were no differences in angular error or response time between participants who reported mental simulation strategies versus abstract strategies in any condition of either Experiment 1 (*t*(73) < 1.26, *p* > .20 in all cases), or Experiment 2 (*t*(81) < .83, *p* > .14 in all cases).

**Table 2:** Each cell shows the number of participants that reported using a particular strategy type (abstract or mental simulation) as a function of cue type for Experiments 1 and 2

Cue type	Experiment 1		Experiment 2	
	Abstract	Mental simulation	Abstract	Mental simulation
Control	3	77	10	73
Arrow	5	37	5	34
Human figure	0	38	4	40

## General discussion

The aims of the current research were to examine (1) how (i.e., at which mental steps) cues such as human figures and arrows affect perspective taking and (2) the relative effects of a human figure versus an arrow in an abstract map-like display, as used in the SOT. Results from two experiments suggest that cues primarily affect step 1 of the perspective-taking process, that is, the process of imagining oneself in the display. They also suggest that a directional cue alone is sufficient to increase response speed on the SOT. Moreover, with the more consistent wording in Experiment 2, directional cues also reduced angular error. The arrow and human figure differ in agency but both provide a directional cue, compared to all other objects in the display (which were chosen to have no directionality). These results support the directionality hypothesis and are contrary to the agency hypothesis (and the results of Gunalp et al., 2019).

Directional cues reduced response time (and angular error in Experiment 2) across all trials, but did not interact with amount of perspective shift or pointing direction. Strategy reports in both experiments suggested that the majority of participants used an embodied strategy to imagine themselves in the array. It appears that the presence of a directional cue primarily affected the step of imagining oneself in the array, which is step 1 of the perspective-taking process.

As predicted, smaller perspective shifts were easier and faster than larger shifts, suggesting either an analog process (c.f., Shepard & Metzler, 1971), a conflict between the participant’s physical and imagined reference frames (de Vega & Rodrigo, 2001; Wraga, 2003), or both. However, perspective shift did not interact with cue condition, suggesting that directional cue does not affect this perspective-taking process (step 2 of the process).

The pointing quadrant accuracy data are suggestive of both an analog process and an advantage for pointing to the front quadrant. Specifically, in both experiments, angular error was smaller when pointing to the front than to the side (left/right quadrants) and to the back, with no difference between side and back, replicating previous findings (Horn & Loomis, 2004; Kozhevnikov & Hegarty, 2001; Shelton & McNamara, 2001). However, response times in both experiments were more consistent with an analog process, in which front was faster than left/right, and both of these were faster than pointing to the back. The discrepancy between the accuracy and response time data for pointing quadrant regarding the mental processes at play during this task indicates that more research is needed to fully grasp the nuanced nature of this task. Again, these patterns were evident regardless of cue, suggesting that directional cue does not affect step 3 of the perspective-taking process.

In interpreting the main effect of cue, it should be noted that both the arrow and the human figure provide a consistent starting point across trials. The arrow and the human figure moved locations within the array on each trial, so there was still some updating required in these conditions. However, participants could always imagine standing at the arrow or person, and assume the direction of that cue. In contrast, in the control condition, participants had to locate a different object on each trial to identify the station point, and locate another object to find the direction to be imagined. Thus, it is possible that performance was enhanced in the human figure and arrow conditions because of consistency of the cue, rather than directionality. For example, the human figure and arrow cue might have become more salient over trials so that attention could be directed more quickly to these cues.

The present findings diverge from those of Gunalp et al. (2019), who found an advantage of a human figure over both arrow and control conditions. This could be because the display used by Gunalp et al. was a more naturalistic scene, the objects (and human figure) were three-dimensional, and the viewing angle was oblique (130° angle) while the display used in the current work was sparse, map-like, and viewed from directly above (180° angle). Additionally, an arrow may be more informative and legible as a directional cue when viewed from directly above than from a side view, while a three-dimensional human figure might be easier to embody than the more abstract figure used here. A comparison of these studies suggests that different directional cues may be effective in different types of displays.

The present work aimed to examine the mechanisms that underlie the benefit in perspective taking when to-be-imagined perspectives are based on a human figure. Results indicated that there were no significant differences between performance with a human figure cue and an arrow cue, both of which benefitted performance in relation to a control condition. The present work provides a valuable theoretical contribution by parsing out steps of the perspective-taking process, allowing for more precise testing of which constituent processes are affected by cues to perspective taking. It indicates the mechanism that underlies the benefit of a human figure or arrow is that it facilitates an embodied process of imagining oneself in the array. This may be the case with other perspective-taking tasks that include agents and arrows. The computerized SOT paradigm used here allows for broad experimental flexibility to test these questions about the underlying nature of perspective taking.

## Footnotes

1 Effects of task order were not significant for either accuracy (*p* = .27) or response time (*p* = .88), so data were collapsed over this factor.

2 Effects of task order were not significant for either accuracy (*p* = .58) or response time (*p* = .65), so data were collapsed over this factor.
